# Prospective evaluation of 92 serum protein biomarkers for early detection of ovarian cancer

**DOI:** 10.1038/s41416-021-01697-z

**Published:** 2022-01-14

**Authors:** Trasias Mukama, Renée Turzanski Fortner, Verena Katzke, Lucas Cory Hynes, Agnese Petrera, Stefanie M. Hauck, Theron Johnson, Matthias Schulze, Catarina Schiborn, Agnetha Linn Rostgaard-Hansen, Anne Tjønneland, Kim Overvad, María José Sánchez Pérez, Marta Crous-Bou, María-Dolores Chirlaque, Pilar Amiano, Eva Ardanaz, Eleanor L. Watts, Ruth C. Travis, Carlotta Sacerdote, Sara Grioni, Giovanna Masala, Simona Signoriello, Rosario Tumino, Inger T. Gram, Torkjel M. Sandanger, Hanna Sartor, Eva Lundin, Annika Idahl, Alicia K. Heath, Laure Dossus, Elisabete Weiderpass, Rudolf Kaaks

**Affiliations:** 1 Division of Cancer Epidemiology, German Cancer Research Center (DKFZ), Heidelberg, Germany; 2 Research Unit Protein Science, Helmholtz Zentrum München, German Center for Environmental Health, Neuherberg, Germany; 3 Department of Molecular Epidemiology, German Institute of Human Nutrition Potsdam -Rehbruecke, Nuthetal, Germany; 4 Institute of Nutritional Science, University of Potsdam, Potsdam, Germany; 5 Danish Cancer Society Research Center, Diet, Genes and Environment, Strandboulevarden 49 DK-2100, Copenhagen, Denmark; 6 Department of Public Health, Aarhus University, Bartholins Alle 2, DK-8000 Aarhus C, Denmark; 7 Escuela Andaluza de Salud Pública (EASP), Granada, Spain; 8 Instituto de Investigación Biosanitaria ibs.GRANADA, Granada, Spain; 9 CIBER in Epidemiology and Public Health (CIBERESP), Madrid, Spain; 10 Department of Preventive Medicine and Public Health, University of Granada, Granada, Spain; 11 Unit of Nutrition and Cancer, Cancer Epidemiology Research Program, Catalan Institute of Oncology (ICO) - Bellvitge Biomedical Research Institute (IDIBELL), L’Hospitalet de Llobregat, Barcelona, 08908 Spain; 12 Department of Epidemiology, Harvard T.H. Chan School of Public Health, Boston, MA 02115 USA; 13 Department of Epidemiology, Regional Health Council, IMIB-Arrixaca, Murcia University, Murcia, Spain; 14 Ministry of Health of the Basque Government, Sub-Directorate for Public Health and Addictions of Gipuzkoa, San Sebastián, Spain; 15 Biodonostia Health Research Institute, Group of Epidemiology of Chronic and Communicable Diseases, San Sebastián, Spain; 16 Navarra Public Health Institute, Pamplona, Spain; 17 IdiSNA, Navarra Institute for Health Research, Pamplona, Spain; 18 Cancer Epidemiology Unit, Nuffield Department of Population Health, University of Oxford, Oxford, OX3 7LF UK; 19 Unit of Cancer Epidemiology, Città della Salute e della Scienza University-Hospital, Via Santena 7, 10126 Turin, Italy; 20 Epidemiology and Prevention Unit, Fondazione IRCCS Istituto Nazionale dei Tumori di Milano, Milano, Italy; 21 Institute of Cancer Research, Prevention and Clinical Network (ISPRO), Florence, Italy; 22 Dipartimento di Salute Mentale e Fisica e Medicina Preventiva, Vanvitelli University, Naples, Italy; 23 Cancer Registry and Histopathology Department, Provincial Health Authority (ASP 7), Ragusa, Italy; 24 Faculty of Health Sciences, Department of Community Medicine, UiT The Arctic University of Norway, N - 9037 Tromsø, Norway; 25 Diagnostic Radiology, Lund University, Department of Medical Imaging and Physiology, Skåne University Hospital, Malmö, Sweden; 26 Department of Medical Biosciences, Pathology, Umeå University, Umeå, Sweden; 27 Department of Clinical Sciences, Obstetrics and Gynecology, Umeå University, Umeå, Sweden; 28 Department of Epidemiology and Biostatistics, School of Public Health, Imperial College London, London, UK; 29 Nutrition and Metabolism Branch, International Agency for Research on Cancer, World Health Organization, Lyon, France; 30 International Agency for Research on Cancer, World Health Organization, Lyon, France

**Keywords:** Diagnostic markers, Ovarian cancer

## Abstract

**Background:**

CA125 is the best available yet insufficiently sensitive biomarker for early detection of ovarian cancer. There is a need to identify novel biomarkers, which individually or in combination with CA125 can achieve adequate sensitivity and specificity for the detection of earlier-stage ovarian cancer.

**Methods:**

In the European Prospective Investigation into Cancer and Nutrition (EPIC) cohort, we measured serum levels of 92 preselected proteins for 91 women who had blood sampled ≤18 months prior to ovarian cancer diagnosis, and 182 matched controls. We evaluated the discriminatory performance of the proteins as potential early diagnostic biomarkers of ovarian cancer.

**Results:**

Nine of the 92 markers; CA125, HE4, FOLR1, KLK11, WISP1, MDK, CXCL13, MSLN and ADAM8 showed an area under the ROC curve (AUC) of ≥0.70 for discriminating between women diagnosed with ovarian cancer and women who remained cancer-free. All, except ADAM8, had shown at least equal discrimination in previous case-control comparisons. The discrimination of the biomarkers, however, was low for the lag-time of >9–18 months and paired combinations of CA125 with any of the 8 markers did not improve discrimination compared to CA125 alone.

**Conclusion:**

Using pre-diagnostic serum samples, this study identified markers with good discrimination for the lag-time of 0–9 months. However, the discrimination was low in blood samples collected more than 9 months prior to diagnosis, and none of the markers showed major improvement in discrimination when added to CA125.

## Background

Current strategies for epithelial ovarian cancer (EOC) screening use a combination of blood-based biomarkers, notably cancer antigen 125 (CA125; mucin 16 (MUC16)) and human epididymis protein (HE)-4, and trans-vaginal ultrasound imaging. However, findings from randomised screening trials and prospective population cohorts have shown insufficient sensitivity and specificity of CA125 and HE4—the currently best two available markers—for the detection of early-stage ovarian tumours [1–5]. Thus, substantial effort is being directed to the search for additional protein biomarkers which either individually or in combination with CA125 and other markers could enhance the sensitivity and specificity for detecting ovarian cancer at an earlier, more treatable stage.

Olink® Proteomics has developed a technology based on the proximity extension assay (PEA) [6, 7], which permits the simultaneous measurement of up to 92 proteins in microliter volumes of blood serum or plasma. Several recent studies have used this multiplex platform to identify biomarkers for ovarian cancer detection, measuring candidate markers in blood samples collected from patients with clinically manifest ovarian cancer and from healthy controls or patients with benign pelvic conditions [8–12]. Using various (only partially overlapping) Olink® assay panels for sets of proteins relevant in oncology, inflammation and other disease areas, these studies identified several candidate proteins that, alone or in multi-marker panels, showed good discrimination between ovarian cancer patients and women with benign conditions or healthy controls. All studies, however, were based on classical case-control comparisons between serum or plasma samples from patients with clinically manifest (and mostly advanced-stage) ovarian cancer and cancer-free control subjects, and so far, there have been no studies examining these markers in blood samples collected from women prior to known cancer, and whether they may help increase the lead time for detection of ovarian cancer.

We here present findings from the first prospective study to evaluate the discriminatory performance of 92 oncology-related protein markers (Olink® Proseek Multiplex Oncology II panel) as potential early diagnostic biomarkers of ovarian cancer. We assessed the ability of the proteins to distinguish women having a future diagnosis of ovarian cancer from healthy controls using measurements from serum samples collected up to 18 months prior to diagnosis. Discrimination capacity is examined within strata of lag-time (0–9 months; >9–18 months) between blood draw and ovarian cancer diagnosis. For markers showing significant differences between case and controls, we corroborate findings through comparisons with the results from previous studies that used similar technology.

## Study setting and methods

### Case-control study, nested within the EPIC cohort

We conducted a case-control study nested within the European Prospective Investigation into Cancer and Nutrition (EPIC) cohort—a population-based, multi-center prospective cohort study in 10 European countries coordinated by the International Agency for Research on Cancer (IARC; Lyon, France) [13]. From 1992 to 2000, 366,521 women were enrolled and of these, 226,673 women provided a blood sample at baseline.

The present work is an extension of an earlier study on the prospective discriminatory capacity of CA125 and other early detection markers for ovarian cancer [4]. It includes pre-diagnostic serum samples from all incident cases (*N* = 91) of epithelial invasive ovarian (International Classification of Diseases for Oncology (ICD-O) code: C569), fallopian tube (C570) or peritoneal cancers (C480, C481, C482 and C488) with available data on tumour histology and diagnosed within maximally 18 months of blood draw (Table 1). All ovarian cancer cases were ascertained prospectively through record linkage with cancer and pathology registries (all countries except France, Germany, Greece, and Naples, Italy), or through active follow-up and systematic verification of self-reports by detailed examination and coding of clinical records (France, Germany and Naples, Italy). Information on tumour stage was available in part from pathology reports and in part from cancer registries, and for uniformity was coded into either local disease (stage I) or high-stage disease (regionally spread or metastatic). Information on tumour characteristics (histologic subtype [serous, endometrioid, clear cell, mucinous, not otherwise specified (NOS)]) and grade was additionally obtained from pathology reports.

**Table 1:** Characteristics of ovarian cancer cases and controls included in the study [median (min–max) or *n* (%)].

Characteristic	Cases (*n* = 91)	Controls (*n* = 182)
Age at blood draw, years	58 (30.3–76.3)	57.8 (30.4–76.3)
Age at blood draw, years		
<50	21 (23%)	39 (21%)
50–54	11 (12%)	24 (13%)
55–59	21 (23%)	50 (27%)
60–64	25 (27%)	44 (24%)
≥65	13 (14%)	25 (14%)
Menopausal status		
Pre	19 (21%)	38 (21%)
Peri^a^	63 (69%)	126 (69%)
Post	9 (10%)	18 (10%)
BMI	24.8 (17.7–40.8)	24.8 (16.9–45.1)
Smoking^b^		
Never	60 (67%)	113 (63%)
Former	13 (15%)	34 (19%)
Current	16 (18%)	31 (17%)
Age at diagnosis	58.6 (30.6–77.6)	–
Lag-time (years)	0.8 (0–1.5)	–
Lag-time (months)		
0–≤9 months	39 (43%)	–
9–≤18 months	52 (57%)	–
Tumour site		
Ovary	88 (97%)	–
Fallopian tube	2 (2%)	–
Peritoneum	1 (1%)	–
Histology		
Serous	54 (59%)	–
Non-serous	37 (41%)	–
Mucinous	10 (11%)	
Endometrioid	7 (8%)	–
Clear cell	4 (4%)	–
NOS	15 (16%)	–
Other	1 (1%)	–
Disease stage^c^		
Localised (Stage I)	19 (21%)	–
Regional (Stage II)	11 (12%)	–
Metastatic (Stage III)	57 (63%)	–
Cancer grade^d^		
Well differentiated	11 (17.7%)	
Moderately differentiated	23 (37.1)	
Poorly differentiated/undifferentiated	28 (45.2)	

^a^Defined as women 42–52 years who have missing or incomplete questionnaire data, reported irregular menstrual cycles in the past 12 months or had a prior hysterectomy without oophorectomy.

^b^Data were missing on smoking for 2 cases and 4 controls.

^c^Data were missing on disease stage at diagnosis for 4 cases.

^d^Data on cancer grade were missing for 29 cases.

For each of the 91 case subjects, two control participants (*N* = 182) were randomly selected among appropriate risk sets consisting of all female cohort members with a blood sample, alive and free of cancer at the time of diagnosis of the index case. An incidence density sampling protocol was used, such that, in principle, control participants could include women who became a cancer case later in time and each control participant could be sampled more than once; however, no control was actually drawn more than once and none of the control participants have subsequently been identified as ovarian cancer cases. Case and control participants were matched on study recruitment centre, age at blood draw (±6 months), time of the day of blood collection (±1 h), fasting status at blood collection, menopausal status at blood collection (premenopausal, perimenopausal, postmenopausal), current use of oral contraceptives or postmenopausal hormone replacements at the time of blood draw and phase of menstrual cycle for premenopausal women (3–5 categories; menstrual phase, follicular phase, ovulatory phase, luteal phase, or unknown, depending on available data).

### Laboratory assays

The Proseek Multiplex Oncology II panel assays were performed in an Olink® certified laboratory at the German Center for Environmental Health (Helmholtz Zentrum München), Neuherberg, Germany. The proximity extension assay (PEA) technology, commercialised by Olink® Proteomics (Uppsala, Sweden), is a highly specific antibody-based technology that allows for relative quantification of numerous human protein biomarkers in body fluids [7]. Serum samples were analysed in batches, sorted by study centre and with samples from matched case-control sets together (in randomised and blinded order) in the same batch. The laboratory personnel were blinded regarding case-control status of the samples analysed. Results of the assays are reported in arbitrary units called ‘normalized protein expression’ values (NPX), which are relative protein expression levels from RT -qPCR on a log2 scale. For small proportions of study subjects, and for a few proteins, PEA measurements fell below the detection limit (IL6 [15%], FADD [26%)] CTSV [3%], MIC-A/B [2%] and CEACAM5 [18%]). When assay results were below the limit of detection, we assigned values to the midpoint between zero and the lower limit of detection.

### Statistical analyses

We used unconditional logistic regression modelling for the estimation of covariate-adjusted receiver operating characteristic (ROC) curves, with calculation of area under curve (AUC) as an overall measure of the markers’ capacity to discriminate future cancer cases from participants who remained ovarian cancer-free. Models were systematically adjusted for study centre, age, menopausal status and use of hormone replacement therapy (HRT) at the time of blood draw as covariates. Analyses focused first on single markers. All models were fit for diagnosis of ovarian cancer of any histologic subtype as outcome and based on these models, individual ovarian cancer risk scores were calculated. The discrimination capacity of the overall risk scores was then further examined by strata of lag-time (≤9 months, >9–18 months) and by histologic subtype (serous vs non-serous or undetermined). Internal validation with 1000‐fold bootstrapping was used to adjust estimates of discriminative capacity for over‐optimism as a result of model overfitting. For all markers showing AUCs ≥0.70, we additionally estimated the sensitivity at 95% and at 98% specificity at cut-off points determined in our datasets for all women who remained cancer-free (*N* = 182).

For markers that showed discrimination of AUC ≥ 0.70 in the 0–9 months lag-time interval, and which had been highlighted as having discrimination potential in at least one previous study (Supplementary Table s1) based on clinical case-control comparisons, we further tested combined discrimination capacity jointly with CA125, using a two-marker discrimination model. These models were first fitted on the full dataset of 91 ovarian cancer cases and 182 controls, covering all lag-times from 0 to 18 months and risk scores were derived based on these models. The discrimination performance of the risk scores was then evaluated by strata of lag-time and histologic subtype. Likelihood-ratio tests were used to test whether the two-marker models significantly improved statistical model fit (and hence discrimination) compared to a model based on MUC16/CA125 only. We performed further analyses to examine the joint discrimination of two-marker model risk scores for tumours diagnosed within 0–9 or >9–18 months after blood draw, as well as by serous or other tumour histology, again using bootstrapping to adjust for over-optimism.

We also investigated whether in more exploratory approach that included all the 92 biomarkers irrespective of their univariate classification power, we could identify markers or panel of markers for discriminating between cases and women who remained cancer-free. This analysis included all women who remained cancer-free and those who were diagnosed with ovarian cancer 9–18 months after blood draw. It is among these women that the discrimination strength of CA125 relative to other marker candidates is not so dominant and improvements in discrimination are likely to be clinically relevant. As biomarkers are expected to be correlated, we used a least absolute shrinkage and selection operator (LASSO) algorithm implemented with the *glmnet* package in R [14] to select a parsimonious model for predicting ovarian cancer diagnosis. All analyses were conducted in SAS, version 9.4 (SAS Institute, Cary, NC) and R version 4.1.0 and RStudio version 1.4.1717 (The R Foundation for Statistical Computing).

## Results

The characteristics of the ovarian cancer cases and matched controls are presented in Table 1. Of the 91 cases, 51 (56%) had serous tumours, whereas the remaining cases had tumours classified as mucinous (*N* = 10; 11%), endometroid (*N* = 7; 8%) clear cell (*N* = 4%) or tumours of non-specified type (NOS, *N* = 15; 16%). The majority (76.5%) of serous ovarian cancers were diagnosed at late stage (Stage III) while half (50%) of the non-serous cancers were at late stage. The distribution of disease spread and grade at diagnosis by histologic subtype is presented in Supplementary Table s2.

**Fig. 1:**
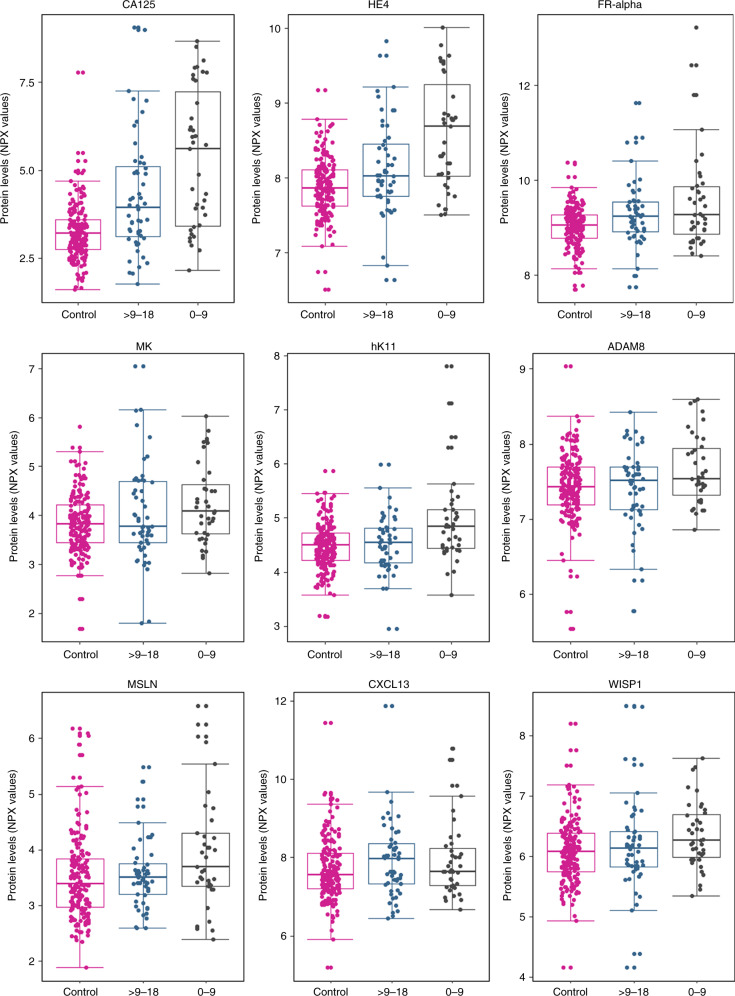
Distributions of protein biomarker levels in controls, and in ovarian cancer. Each of the panels shows marker distributions in the form of Box plots, for biomarkers that yielded an AUC ≥ 0.7 for ovarian cancer diagnosis 0–9 months after blood draw. For the cancer cases, the plots show marker distributions measured in blood samples that had been collected 0–9 or >9–18 months prior to diagnosis.

Nine biomarkers had an AUC of at least 0.7 for discriminating between women who developed ovarian cancer and women who remained ovarian cancer-free for at least 9 months after blood draw (Supplementary Table s3). The distribution of the levels of the nine markers in controls and in cases (by lag-time) are presented in Fig. 1. After performing 1000-fold bootstrapping to correct for potential over-optimism, all the nine markers maintained at least an AUC of 0.7 for distinguishing between controls and ovarian cancer cases diagnosed within 9 months of blood draw (Table 2). Of the 9 biomarkers, 8 had been previously reported to be informative for discrimination in at least one previous study comparing between ovarian cancer patients and healthy controls or patients with benign tumours (for an overview of the previous study findings, see Supplementary Table s1). CA125 (MUC16) and HE4 (WFDC2) had the highest discrimination with AUCs of 0.77 (95% CI: 0.75–0.79) and 0.73 (95% CI: 0.71–0.74), respectively, for the entire time lag of 0–18 months. The other markers that had been highlighted at least twice in previous studies and had good discrimination between healthy women and women who were diagnosed with either serous or non-serous ovarian cancer within 9 months of blood collection in the current study were: folate receptor alpha (FOLR1/FR-alpha), kallikrein (KLK11), midkine (MDK/MK) and C-X-C motif chemokine 13 (CXCL13). Two other markers—WNT1-inducible signaling pathway protein 1 (WISP1) and mesothelin (MSLN) had been found to have discriminatory potential in at least one cross-sectional case-control comparison (Table 2).

**Table 2:** Over-optimism corrected diagnostic performance of single markers, by lag-time and ovarian cancer histology.

	Lag-time (months)																	
	0–18						0–9						9–18					
	All		Serous		Non-serous		All		Serous		Non-serous		All		Serous		Non-serous	
Protein	AUC	95% CI	AUC	95% CI	AUC	95% CI	AUC	95% CI	AUC	95% CI	AUC	95% CI	AUC	95% CI	AUC	95% CI	AUC	95% CI
MUC16 [8–12]	0.77	0.75–0.79	0.83	0.80–0.85	0.69	0.65–0.72	0.84	0.81–0.87	0.86	0.82–0.89	0.82	0.76–0.87	0.72	0.69–0.74	0.80	0.76–0.84	0.61	0.55–0.65
WFDC2 [8–12]	0.73	0.71–0.74	0.78	0.75–0.80	0.66	0.63–0.68	0.86	0.82–0.89	0.88	0.83–0.91	0.82	0.75–0.87	0.63	0.61–0.66	0.70	0.65–0.74	0.56	0.51–0.59
FOLR1 [8, 10–12]	0.66	0.64–0.69	0.71	0.68–0.74	0.60	0.56–0.64	0.73	0.69–0.77	0.79	0.75–0.84	0.61	0.54–0.68	0.62	0.59–0.64	0.64	0.60–0.68	0.60	0.54–0.64
KLK11 [9–11]	0.61	0.59–0.63	0.66	0.63–0.69	0.54	0.50–0.57	0.73	0.69–0.77	0.78	0.73–0.83	0.65	0.57–0.71	0.52	0.49–0.56	0.57	0.52–0.62	0.53	0.48–0.59
MDK [8–11]	0.61	0.59–0.63	0.65	0.62–0.69	0.55	0.51–0.58	0.67	0.63–0.72	0.71	0.65–0.78	0.60	0.54–0.66	0.56	0.53–0.59	0.60	0.55–0.65	0.51	0.47–0.55
WISP1 [8]	0.61	0.59–0.63	0.62	0.59–0.65	0.59	0.54–0.63	0.73	0.68–0.76	0.72	0.66–0.77	0.74	0.67–0.80	0.52	0.49–0.55	0.54	0.50–0.59	0.51	0.47–0.56
CXCL13 [8, 9]	0.59	0.58–0.61	0.54	0.49–0.57	0.68	0.63–0.71	0.63	0.59–0.66	0.58	0.52–0.62	0.71	0.65–0.77	0.57	0.55–0.59	0.50	0.46–0.57	0.65	0.59–0.70
MSLN [10]	0.59	0.57–0.60	0.60	0.57–0.63	0.57	0.54–0.60	0.67	0.64–0.69	0.74	0.70–0.77	0.55	0.48–0.61	0.53	0.48–0.55	0.52	0.48–0.56	0.58	0.54–0.61
ADAM8	0.56	0.53–0.58	0.58	0.54–0.61	0.53	0.49–0.57	0.68	0.63–0.71	0.75	0.67–0.80	0.56	0.49–0.62	0.53	0.50–0.56	0.56	0.52–0.61	0.51	0.47–0.56

n—studies using Olink® proximity extension assays that have showed the marker to have good discrimination.

All the nine best-performing biomarkers showed a decay in discrimination strength with longer lag-time between blood draw and ovarian cancer diagnosis (Table 2). For instance, there were five markers with AUCs of at least 0.7 for discriminating between healthy women and ovarian cancer cases (all histologies) for the lag-time of 0–9 months but only CA125 had an AUC higher than 0.7 for the lag-time of >9–18 months. As a second example, the discrimination performance of FR-alpha was 0.73 (95% CI: 0.69–0.77) for the lag-time of 0–9 months and only 0.62 (95% CI: 0.59–0.64) for the lag-time of 9–18 months. Similar reductions in discrimination performance with longer lag-time between blood draw and diagnosis were observed for all other markers. HE4/WFDC2 had an AUC of 0.63 (95% CI: 0.61–0.66) for the lag-time of 9–18 months (Table 2). Based on their sensitivities at 95% and at 98% specificity, the nine markers showed a similar decay in performance (Table 3). Within the lag period of 0–9 months, most markers showed stronger discrimination for serous tumours as compared to tumours with other or unspecified histology, with notable exceptions for WISP1, and CXCL13 which showed higher AUCs for the tumours of non-serous histology.

**Table 3:** Sensitivity at 95% and 98% specificity for the top biomarkers by time between blood draw and ovarian cancer diagnosis.

	Sensitivity at 95% specificity by lag-time (months)			Sensitivity at 98% specificity by lag-time (months)		
Marker	0–≤9	9–≤18	0–≤18	0–≤9	9–≤18	0–≤18
CA125	0.54 (0.34–0.72)	0.37 (0.21–0.55)	0.44 (0.29–0.60)	0.51 (0.30–0.73)	0.37 (0.21–0.57)	0.27 (0.13–0.48)
HE4	0.51 (0.32–0.70)	0.21 (0.10–0.39)	0.34 (0.21–0.50)	0.49 (0.27–0.70)	0.31 (0.16–0.50)	0.17 (0.07–0.37)
FR-alpha	0.33 (0.18–0.54)	0.19 (0.09–0.36)	0.25 (0.14–0.41)	0.26 (0.11–0.49)	0.19 (0.08–0.36)	0.13 (0.05–0.32)
MK	0.21 (0.09–0.40)	0.13 (0.06–0.29)	0.16 (0.08–0.30)	0.18 (0.07–0.40)	0.15 (0.07–0.32)	0.13 (0.05–0.32)
CXCL13	0.15 (0.06–0.34)	0.13 (0.06–0.29)	0.14 (0.07–0.27)	0.10 (0.03–0.30)	0.07 (0.02–0.19)	0.04 (0.01–0.18)
hK11	0.23 (0.11–0.43)	0.06 (0.02–0.19)	0.13 (0.06–0.26)	0.15 (0.05–0.37)	0.09 (0.03–0.23)	0.04 (0.01–0.18)
ADAM8	0.18 (0.08–0.37)	0.08 (0.02–0.22)	0.12 (0.06–0.24)	0.15 (0.05–0.37)	0.08 (0.03–0.21)	0.02 (0.00–0.16)
WISP1	0.13 (0.05–0.31)	0.10 (0.03–0.24)	0.11 (0.05–0.23)	0.08 (0.02–0.26)	0.08 (0.03–0.21)	0.08 (0.02–0.24)
MSLN	0.15 (0.06–0.34)	0.04 (0.01–0.16)	0.09 (0.04–0.20)	0.10 (0.03–0.30)	0.04 (0.01–0.15)	0.00 (0.00–0.07)

**Fig. 2:**
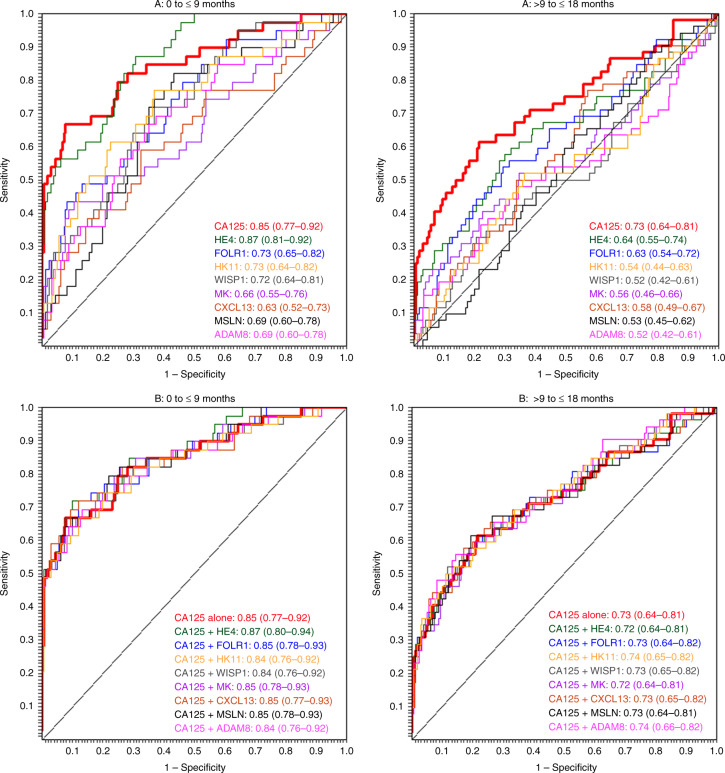
ROC plots for top biomarkers individually and in combination with CA125, by lag-time. Top row (**a**) shows the performance of individual biomarkers. Bottom row (**b**) shows the performance of the biomarkers when combined with CA125.

For all the eight markers that individually had AUCs ≥ 0.70, we examined the discrimination potential of the markers alone and in combination with CA125 (Table 4; Fig. 2). Using the likelihood-ratio test (LRT), we tested improvement in model fit of adding any of the eight markers to a model containing CA125 alone. None of the biomarkers resulted in significant improvements in model fit. We also observed only minor improvements in the corresponding joint discrimination with magnitude of 1%-point increase in discrimination strength compared to CA125 alone. Subtle improvements in discrimination were noted in further analysis by lag-time and histology. For instance, combination of CA125 and HE4 had a 1%-point higher performance than CA125 alone for all ovarian cancer histologies combined, for the lag-time of 0–9 months. Similarly, for the lag-time of >9–18 months, a combination of CA125 with ADAM8 had a slightly better performance, by 2% points than CA125 alone (Table 3). All the eight biomarkers were positively correlated with CA125 (Supplementary Fig. 1).

**Table 4:** Diagnostic performance of CA125 (plus one biomarker) for distinguishing between serum from ovarian cancer patients and healthy controls by lag-time and histology.

	Lag-time (months)																	
	0–18						0–9						9–18					
	All		Serous		Non-serous		All		Serous		Non-serous		All		Serous		Non-serous	
Proteins	AUC	95% CI	AUC	95% CI	AUC	95% CI	AUC	95% CI	AUC	95% CI	AUC	95% CI	AUC	95% CI	AUC	95% CI	AUC	95% CI
CA125 alone	0.77	0.75–0.79	0.83	0.80–0.85	0.69	0.65–0.72	0.84	0.81–0.87	0.86	0.82–0.89	0.82	0.76–0.87	0.72	0.69–0.74	0.80	0.76–0.84	0.61	0.55–0.65
CA125 + ADAM8	0.78	0.76–0.80	0.83	0.81–0.85	0.70	0.66–0.74	0.83	0.80–0.87	0.84	0.79–0.88	0.82	0.77–0.87	0.74	0.70–0.77	0.83	0.78–0.87	0.63	0.57–0.68
CA125 + CXCL13	0.78	0.75–0.79	0.81	0.78–0.84	0.72	0.66–0.76	0.84	0.81–0.88	0.85	0.81–0.89	0.84	0.78–0.89	0.72	0.70–0.75	0.78	0.73–0.83	0.65	0.57–0.70
CA125 + FOLR1	0.77	0.75–0.79	0.83	0.80–0.85	0.70	0.65–0.73	0.85	0.80–0.88	0.86	0.82–0.91	0.82	0.76–0.87	0.72	0.69–0.75	0.80	0.76–0.84	0.62	0.56–0.67
CA125 + KLK11	0.77	0.75–0.79	0.83	0.80–0.85	0.69	0.65–0.72	0.83	0.79–0.87	0.85	0.81–0.89	0.80	0.73–0.86	0.73	0.69–0.76	0.81	0.77–0.85	0.62	0.57–0.68
CA125 + MDK	0.77	0.75–0.79	0.82	0.79–0.85	0.69	0.65–0.73	0.84	0.81–0.87	0.86	0.82–0.89	0.82	0.76–0.87	0.71	0.68–0.74	0.79	0.74–0.83	0.62	0.56–0.66
CA125 + MSLN	0.77	0.75–0.79	0.83	0.80–0.85	0.69	0.65–0.72	0.85	0.81–0.88	0.87	0.83–0.91	0.81	0.75–0.87	0.71	0.69–0.74	0.79	0.75–0.84	0.62	0.56–0.66
CA125 + HE4	0.77	0.75–0.79	0.83	0.80–0.85	0.69	0.65–0.72	0.86	0.81–0.90	0.87	0.83–0.91	0.84	0.77–0.89	0.71	0.68–0.74	0.80	0.75–0.84	0.61	0.55–0.65
CA125 + WISP1	0.77	0.75–0.79	0.83	0.80–0.85	0.69	0.65–0.72	0.84	0.80–0.88	0.86	0.81–0.89	0.81	0.74–0.87	0.72	0.69–0.75	0.80	0.75–0.84	0.62	0.56–0.66

To further explore whether any marker combinations exited that might contribute discrimination information beyond that provided by CA125 alone, we conducted further analyses using a more exploratory approach including all 92 markers. The analyses employed a LASSO algorithm for model selection and included cases diagnosed 9–18 months following blood draw and all women who remained cancer-free. We found that the most regularised parsimonious model for predicting case-control status of ovarian cancer, at value of regularisation parameter (λ) such that the cross-validated error is within one standard error of the minimum, i.e. lambda.1se, contained only CA125.

## Discussion

Using serum samples and data collected in the EPIC cohort, we examined the capacity of 92 cancer-related protein biomarkers measured using the Olink® Proteomics Oncology II panel to discriminate between women who were prospectively diagnosed with ovarian cancer and matched control women who remained cancer-free. In the analysis of women who developed ovarian cancer less than 9 months after blood draw, nine biomarkers showed potentially useful discrimination, with AUC ≥ 0.70. Besides the well-established markers CA125 and HE4, four other markers in this list: (FR-alpha, KLK11, MDK and CXCL13) had been highlighted previously as having discrimination potential in several prior case-control comparisons using the Olink® Multiplex platforms [8–12, 15], as well as in some further studies using other platforms [16–18]. We did not observe meaningful improvements in diagnostic performance by adding a single markers to CA125, particularly for ovarian cancer diagnosed more than 9 months after blood sampling.

Eight of the nine best-performing biomarkers had been previously reported by at least one case-control study to show good discrimination between ovarian cancer cases and controls. This concordance with earlier findings suggests that the proteins could be genuinely associated with cancer development and indeed, most of the biomarkers have been implicated to play a role in ovarian carcinogenesis or associated with ovarian cancer prognosis. FOLR1/FR-alpha is involved in the unidirectional transportation of folates into cells, metabolism of which facilitates DNA synthesis, methylation and repair [19]. In normal ovarian tissue, the expression of FOLR1 is restricted to luminal surfaces but is ubiquitous in ovarian tumour tissue, mostly in tumours of non-mucinous histology [20]. CXCL13 has been shown to play a role in immune cell recruitment to the site of chronic inflammation, activation and adaptive immune response regulation [21]. KLK11/hK11 was reported to be highly expressed in ovarian cancer patients, mostly early-stage tumours and is thus a potential marker of favourable prognosis [22]. WISP1 is believed to play a role in a number of cancers and is associated with poor survival and clinical grades of endometrial adenocarcinoma (endometrioid type) [23, 24].

The diagnostic performance of CA125, HE4 and other candidate biomarkers on the panel markedly reduced with increasing lag-time between blood draw and cancer diagnosis, which is in line with previous studies [3, 4, 25], and is to be expected for markers genuinely associated with tumour development. However, to be beneficial, markers or marker combinations should provide sufficient early detection lead time, such that earlier medical intervention can improve a patient’s survival. Previous studies suggested that, while at least for some patients serum biomarker levels indicative of ovarian cancer could be detectable at most 3 years prior to diagnosis, for most patients the likely lead time was less than 1 year [3, 4, 25–27]. We found that other than CA125, none of the other markers provided useful discriminatory information for ovarian cancer detected more than 9 months after blood draw and that paired combinations of CA125 with any other of the 8 markers did not meaningfully improve discrimination compared to CA125 alone, in either the 0–9 or the >9–18 months lag-time intervals. Similarly, in a more exploratory approach using LASSO algorithms that sought to identify markers or panel of markers for discriminating between cases and controls in samples collected 9–18 months prior to diagnosis, and regardless of the univariate classification power of the biomarker or being previously shown to have discrimination potential, we still could not identify markers that improved on the discrimination of CA125, without model overfitting. Thus, given that CA125 is the best available but insufficiently sensitive marker of ovarian cancer, our findings suggest that none of the biomarkers investigated in this study has sufficient potential to extend the lead time longer than that provided by CA125 alone.

Our study has some limitations. For women who were diagnosed with ovarian cancer, we have no knowledge about the stage of the tumours at the time of blood draw, making it difficult to speculate whether improved discrimination performance of the markers would result in survival benefit. Also, as we examined a total of 92 markers, our analyses may have resulted in false-positive leads observed only by chance, although most of the markers with AUC > 0.7 (0–9-month interval) in our present dataset had shown discrimination potential in previous case-control studies including prevalent cases. Conversely, due to the limited sample size it is possible that some markers truly associated with ovarian cancer were missed. When testing two-marker combinations (CA125 plus any other marker) we observed no meaningful improvements in discrimination. Developing accurately weighted marker scores for more than two markers (more than one additionally to CA125) will require larger numbers of ovarian cancer cases than in our present study and could be achieved by combining serum samples and data of ovarian cancer cases and matched control subjects from additional large-scale population cohorts worldwide.

## Conclusion

Our study confirms the good discrimination between ovarian cancer cases and controls of several biomarkers previously observed in cross-sectional studies. However, markers showed discrimination only in samples collected 9 months prior to ovarian cancer diagnosis and much less so in samples collected 9–18 months prior to diagnosis. Unfortunately, combining single markers with CA125 did not improve the diagnostic performance of the markers.

## Disclaimer

Where authors are identified as personnel of the International Agency for Research on Cancer/World Health Organization, the authors alone are responsible for the views expressed in this article and they do not necessarily represent the decisions, policy or views of the International Agency for Research on Cancer/World Health Organization.

## Supplementary information

Supplementary Figure 1

Supplementary Tables

## Data Availability

The EPIC project was launched in the 1990s. Unlike in new studies that we run today, public access to data from the EPIC population was not part of the study protocol at that time. Thus, the data protection statement and informed consent of the EPIC participants do not cover the provision of data in public repositories. Nevertheless, we are open to provide our dataset upon request for (a) statistical validation by reviewers and (b) pooling projects under clearly defined and secure conditions and based on valid data transfer agreements.
