# Supplementary material for: Prospective evaluation of 92 serum protein biomarkers for early detection of ovarian cancer
Source: Br J Cancer. 2022 Jan 14;126(9):1301–9. doi: 10.1038/s41416-021-01697-z (PMC9042845; doi:10.1038/s41416-021-01697-z)
Supplement: Supplementary file 1 — Supplementary Figure 1 [file 41416_2021_1697_MOESM1_ESM.docx]

**
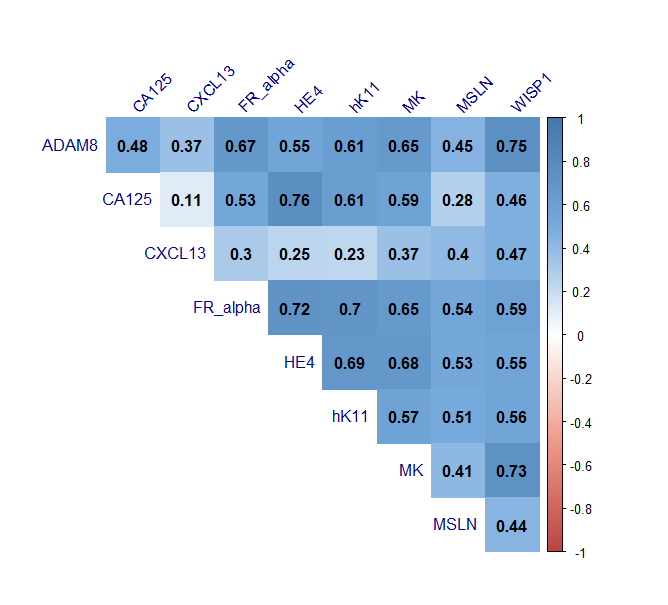
**

**Supplementary Figure 1.** Correlation matrix for concentrations of the 9 protein biomarkers in cases.
