# Supplementary material for: Prospective evaluation of 92 serum protein biomarkers for early detection of ovarian cancer
Source: Br J Cancer. 2022 Jan 14;126(9):1301–9. doi: 10.1038/s41416-021-01697-z (PMC9042845; doi:10.1038/s41416-021-01697-z)
Supplement: Supplementary file 2 — Supplementary Tables [file 41416_2021_1697_MOESM2_ESM.docx]

**Supplementary Table s1.** Studies using proximity extension assay platforms and the identified candidate biomarkers that overlap with the Proseek Oncology II panel used in the study.

| **Author, year** | **Sample size** | **Samples used** | **Histology** | **Panel(s) used** | **Criteria** | **Identified biomarkers that overlap with the panel used in this study** |
| --- | --- | --- | --- | --- | --- | --- |
| Leandersson et.all, 2020[1] | 180 women with benign, borderline, or epithelial ovarian cancer. | Plasma | All | Olink® Oncology II and Inflammation panels | Identified 34 biomarkers with AUC≥0.7 for distinguishing between benign tumours and cancer | WFDC2/HE4, MUC16/CA125, FOLR1/FR-alpha, IL6, SCF, PVRL4/Nectin-4, MDK/MK, WISP1, RSPO3, TFPI2, CXCL13, VEGFR2, TNFRSF4, ITGAV, TNFRSF6B, PVRL4, ABL1, VEGFA, ABL1, and CTSV |
| Skubitz et.al 2019[2] | 61patients with epithelial ovarian/fallopian tube or primary  peritoneal cancer and 88 healthy participants | Serum | Serous | Proseek Oncology II | Identified 40 biomarkers with AUC ≥0.7 for distinguishing sera of cancer patients and healthy women | WFDC2/HE4, MUC16/CA125, FOLR1/FR-alpha, KLK11/hk11, MSLN, MDK/MK, IFNGR1, RSPO3, CD48, CD70, IL6, VEGFR2, ITGAV, KLK13, GPNMB, XPNPEP2, EGF, CD70, RET, LY9, CEACAM5, ADAMTS15, ITGB5, TLR3, FADD, GPC1, ERBB2, MIA, CEACAM1, IFNGR1, S100A4, FGFBP1, ICOSLG, SCF, CD207, FASLG, CRNN, GZMB, LYPD3, and TNFRSF19 |
| Enroth et.al 2019[3] | 90 patients with benign tumours and 79 with ovarian cancer for discovery stage, 71 benign tumours and 100 controls for the first replication, 13 stage I/II and 64 stage III/IV for the second replication and 106 benign tumours, 28 borderline and 87 ovarian cancers for the third replication | Plasma | All | Olink Multiplex CVD II, CVD III, Inflammation, Neurology, Oncology  Cardiometabolic, Cell Regulation, Development,  Immune Response, Metabolism and Organ Damage | Identified best performing and practically usable 11-biomarker model out of 484 candidate models. The 11-marker panel achieved AUC of 0.94 (95%  CI 0.91–0.98) with a sensitivity of 0.85 at a specificity of 0.93. | WFDC2/HE4, MUC16/CA125, FOLR1/FR-alpha, and ADAMTS15 |
| Enroth et.al 2018[4] | 150 benign tumours, 106 ovarian cancer and 399 healthy controls for the discovery step and  76 benign tumours, 160 ovarian cancer and 57 controls for the replication step | Plasma | All | 5 Olink Multiplex assays (CVD II, CVD III, INF I, ONC II, and NEU I) | Identified 13 biomarkers out of 441 candidates based on p-values of association | WFDC2/HE4, MUC16/CA125, FOLR1/FR-alpha, KLK11/hk11, PVRL4/Nectin-4, MDK/MK, and PVRL4 |
| Boylan et.al 2017[5] | 21 healthy controls, 18 benign ovarian disease, and 42 (21 early stage I/II serous and 21 late stage III/IV serous) ovarian cancer | Serum | Serous | The Proseek® Multiplex Oncology  Iv2 | Identified 25 markers with AUCs ≥0.8 for distinguishing late-stage ovarian cancer and healthy women, 25 biomarkers for distinguishing between late stage and benign disease with AUCs ≥0.74, and 25 biomarkers with AUCs ≥0.69 for distinguishing between early-stage ovarian cancer and healthy women. | WFDC2/HE4, MUC16/CA125, FOLR1/FR-alpha, KLK11/hk11, MDK/MK, IL6, CXCL13, EGF, HGF, TGFA, VEGFA, SCF, IFNGR1 and FADD |

**Supplementary Table s2**. Ovarian cancer stage and grade at diagnosis by histologic subtype

|  | **Histology, n (%)** | | |
| --- | --- | --- | --- |
| **Characteristic** | **serous** | **non-serous*** | **All** |
| **Disease spread** |  |  |  |
| Localised (stage I) | 5 (9.3) | 14 (37.7) | 19 (20.9) |
| Regional (stage II) | 7 (13.0) | 4 (10.8) | 11 (12.1) |
| Metastatic (stage III) | 39 (72.2) | 18 (48.7) | 57 (62.6) |
| Missing | 3 (5.5) | 1 (2.7) | 4 (4.4) |
| **Cancer grade** |  |  |  |
| Well differentiated | 5 (9.3) | 6 (16.2) | 11 (12.1) |
| Moderately differentiated | 17 (31.5) | 6 (16.2) | 23 (25.3) |
| Poorly differentiated/undifferentiated | 16 (29.6) | 12 (32.4) | 28 (30.8) |
| Missing | 16 (29.6) | 13 (35.1) | 29 (31.9) |

*includes mucinous, endometrioid, clear cell, NOS and unspecified.

**Supplementary Table s3.** Diagnostic performance of all 92 individual biomarkers on the Olink® Proseek Oncology II by lag-time and histology.

|  | **lag-time (months)** | | | | | | | | | | | | | | | | | | |
| --- | --- | --- | --- | --- | --- | --- | --- | --- | --- | --- | --- | --- | --- | --- | --- | --- | --- | --- | --- |
|  | **All** | | | | | | **0-≤9** | | | | | | | **>9 - ≤18** | | | | | |
|  | **All** | | **Serous** | | **non-serous** | | **All** | | **serous** | | **non-serous** | | **All** | | | **serous** | | **non-serous** | |
| **Protein** | **AUC (95% CI)** | **p** | **AUC (95% CI)** | **p** | **AUC (95% CI)** | **p** | **AUC (95% CI)** | **p** | **AUC (95% CI)** | **p** | **AUC (95% CI)** | **p** | **AUC (95% CI)** | | **p** | **AUC (95% CI)** | **p** | **AUC (95% CI)** | **p** |
| MUC16[1-5] | 0.78(0.72-0.84) | <0.001 | 0.84(0.77-0.91) | <0.001 | 0.69(0.59-0.80) | <0.001 | 0.85(0.77-0.92) | <0.001 | 0.86(0.77-0.95) | <0.001 | 0.82(0.69-0.95) | <0.001 | 0.73(0.64-0.81) | | <0.001 | 0.81(0.72-0.91) | <0.001 | 0.62(0.48-0.75) | 0.097 |
| WFDC2[1-5] | 0.74(0.67-0.80) | <0.001 | 0.79(0.71-0.86) | <0.001 | 0.67(0.56-0.78) | 0.003 | 0.87(0.81-0.92) | <0.001 | 0.89(0.82-0.95) | <0.001 | 0.84(0.74-0.93) | <0.001 | 0.64(0.55-0.74) | | 0.004 | 0.7(0.59-0.81) | <0.001 | 0.56(0.41-0.71) | 0.406 |
| FOLR1[1-4] | 0.67(0.60-0.74) | <0.001 | 0.71(0.63-0.79) | <0.001 | 0.62(0.52-0.72) | 0.020 | 0.73(0.65-0.82) | <0.001 | 0.79(0.68-0.90) | <0.001 | 0.63(0.50-0.77) | 0.050 | 0.63(0.54-0.72) | | 0.006 | 0.64(0.53-0.75) | 0.016 | 0.61(0.48-0.75) | 0.106 |
| KLK11[2,4,5] | 0.62(0.55-0.69) | 0.001 | 0.67(0.58-0.76) | <0.001 | 0.55(0.44-0.66) | 0.393 | 0.73(0.64-0.82) | <0.001 | 0.77(0.66-0.88) | <0.001 | 0.66(0.51-0.82) | 0.040 | 0.54(0.44-0.63) | | 0.464 | 0.58(0.46-0.70) | 0.186 | 0.52(0.38-0.66) | 0.760 |
| ADAM8 | 0.57(0.49-0.64) | 0.078 | 0.58(0.49-0.68) | 0.080 | 0.55(0.43-0.66) | 0.425 | 0.69(0.60-0.78) | <0.001 | 0.76(0.67-0.86) | <0.001 | 0.57(0.40-0.73) | 0.439 | 0.52(0.43-0.62) | | 0.624 | 0.57(0.45-0.70) | 0.252 | 0.46(0.31-0.62) | 0.651 |
| MSLN[2] | 0.6(0.53-0.67) | 0.006 | 0.61(0.52-0.69) | 0.015 | 0.59(0.50-0.67) | 0.060 | 0.69(0.60-0.78) | <0.001 | 0.75(0.66-0.85) | <0.001 | 0.57(0.43-0.71) | 0.318 | 0.53(0.45-0.62) | | 0.459 | 0.52(0.41-0.63) | 0.764 | 0.59(0.49-0.70) | 0.073 |
| WISP1[1] | 0.61(0.53-0.68) | 0.005 | 0.61(0.52-0.70) | 0.018 | 0.6(0.50-0.70) | 0.060 | 0.72(0.64-0.81) | <0.001 | 0.71(0.61-0.82) | <0.001 | 0.74(0.61-0.86) | <0.001 | 0.52(0.42-0.61) | | 0.698 | 0.52(0.39-0.65) | 0.758 | 0.48(0.35-0.62) | 0.798 |
| CXCL13[1-5] | 0.6(0.53-0.67) | 0.006 | 0.54(0.46-0.63) | 0.353 | 0.69(0.58-0.80) | <0.001 | 0.63(0.52-0.73) | 0.015 | 0.58(0.45-0.70) | 0.223 | 0.72(0.55-0.89) | 0.011 | 0.58(0.49-0.67) | | 0.074 | 0.49(0.39-0.59) | 0.878 | 0.67(0.54-0.81) | 0.013 |
| MDK[1,2,4,5] | 0.6(0.53-0.68) | 0.009 | 0.64(0.55-0.73) | <0.001 | 0.54(0.42-0.67) | 0.488 | 0.66(0.55-0.76) | <0.001 | 0.72(0.61-0.83) | <0.001 | 0.59(0.40-0.77) | 0.348 | 0.56(0.46-0.66) | | 0.24 | 0.6(0.48-0.71) | 0.12 | 0.52(0.35-0.68) | 0.846 |
| PODXL | 0.56(0.48-0.63) | 0.133 | 0.61(0.52-0.70) | 0.01 | 0.48(0.37-0.59) | 0.683 | 0.64(0.54-0.74) | 0.007 | 0.69(0.57-0.82) | <0.001 | 0.49(0.33-0.66) | 0.926 | 0.5(0.40-0.59) | | 0.95 | 0.52(0.40-0.64) | 0.75 | 0.53(0.39-0.67) | 0.655 |
| TNFRSF4[1] | 0.56(0.49-0.64) | 0.084 | 0.56(0.47-0.64) | 0.216 | 0.58(0.47-0.69) | 0.160 | 0.66(0.57-0.75) | <0.001 | 0.67(0.57-0.78) | 0.001 | 0.63(0.49-0.78) | 0.072 | 0.51(0.41-0.60) | | 0.906 | 0.55(0.43-0.66) | 0.428 | 0.55(0.40-0.69) | 0.545 |
| PVRL4[1-4] | 0.58(0.50-0.65) | 0.039 | 0.59(0.51-0.68) | 0.035 | 0.56(0.44-0.67) | 0.329 | 0.65(0.56-0.74) | 0.002 | 0.67(0.57-0.78) | 0.002 | 0.61(0.45-0.78) | 0.189 | 0.52(0.43-0.61) | | 0.648 | 0.52(0.41-0.63) | 0.712 | 0.48(0.33-0.62) | 0.764 |
| VEGFR2[2] | 0.56(0.49-0.64) | 0.081 | 0.54(0.45-0.62) | 0.409 | 0.61(0.50-0.71) | 0.050 | 0.62(0.53-0.72) | 0.013 | 0.59(0.47-0.71) | 0.134 | 0.67(0.54-0.81) | 0.012 | 0.52(0.43-0.61) | | 0.628 | 0.51(0.41-0.62) | 0.829 | 0.57(0.42-0.71) | 0.370 |
| RSPO3[1,2] | 0.51(0.43-0.58) | 0.819 | 0.56(0.47-0.64) | 0.199 | 0.56(0.46-0.67) | 0.247 | 0.64(0.55-0.74) | 0.002 | 0.67(0.56-0.78) | 0.002 | 0.6(0.45-0.74) | 0.204 | 0.59(0.51-0.68) | | 0.038 | 0.54(0.43-0.65) | 0.465 | 0.66(0.54-0.78) | 0.011 |
| GZMB[2] | 0.54(0.46-0.61) | 0.333 | 0.52(0.43-0.60) | 0.716 | 0.56(0.46-0.67) | 0.221 | 0.54(0.44-0.64) | 0.402 | 0.52(0.42-0.63) | 0.671 | 0.66(0.49-0.82) | 0.062 | 0.53(0.44-0.62) | | 0.489 | 0.55(0.44-0.66) | 0.383 | 0.51(0.39-0.63) | 0.901 |
| ERBB4 | 0.57(0.49-0.64) | 0.074 | 0.6(0.51-0.69) | 0.036 | 0.53(0.42-0.64) | 0.630 | 0.6(0.50-0.70) | 0.040 | 0.65(0.53-0.77) | 0.012 | 0.52(0.36-0.68) | 0.826 | 0.54(0.44-0.64) | | 0.409 | 0.55(0.43-0.67) | 0.452 | 0.47(0.32-0.61) | 0.658 |
| TNFRSF6B[1] | 0.56(0.48-0.63) | 0.123 | 0.54(0.45-0.63) | 0.390 | 0.59(0.48-0.69) | 0.121 | 0.65(0.56-0.74) | 0.001 | 0.65(0.55-0.76) | 0.004 | 0.65(0.50-0.80) | 0.052 | 0.51(0.42-0.61) | | 0.789 | 0.56(0.44-0.68) | 0.306 | 0.55(0.40-0.69) | 0.518 |
| KLK14 | 0.51(0.44-0.59) | 0.707 | 0.55(0.46-0.63) | 0.278 | 0.54(0.43-0.64) | 0.523 | 0.57(0.47-0.67) | 0.146 | 0.53(0.41-0.66) | 0.608 | 0.65(0.50-0.79) | 0.047 | 0.58(0.49-0.67) | | 0.072 | 0.62(0.52-0.72) | 0.022 | 0.53(0.40-0.67) | 0.626 |
| XPNPEP2[2] | 0.54(0.46-0.61) | 0.335 | 0.56(0.47-0.64) | 0.191 | 0.51(0.39-0.62) | 0.913 | 0.59(0.49-0.68) | 0.077 | 0.55(0.44-0.67) | 0.336 | 0.64(0.48-0.81) | 0.081 | 0.5(0.41-0.59) | | 0.966 | 0.56(0.45-0.67) | 0.310 | 0.42(0.28-0.56) | 0.270 |
| TFPI2[1] | 0.57(0.50-0.65) | 0.063 | 0.58(0.49-0.68) | 0.077 | 0.55(0.44-0.66) | 0.341 | 0.61(0.51-0.71) | 0.039 | 0.64(0.52-0.76) | 0.020 | 0.54(0.38-0.70) | 0.645 | 0.54(0.45-0.64) | | 0.367 | 0.53(0.40-0.66) | 0.630 | 0.56(0.42-0.70) | 0.387 |
| FOLR3 | 0.54(0.47-0.61) | 0.231 | 0.51(0.43-0.60) | 0.735 | 0.59(0.48-0.69) | 0.098 | 0.49(0.40-0.59) | 0.915 | 0.49(0.38-0.61) | 0.930 | 0.51(0.34-0.67) | 0.947 | 0.58(0.50-0.66) | | 0.063 | 0.53(0.43-0.63) | 0.551 | 0.64(0.52-0.76) | 0.020 |
| ERBB3 | 0.54(0.47-0.62) | 0.254 | 0.53(0.44-0.62) | 0.540 | 0.57(0.45-0.68) | 0.255 | 0.56(0.46-0.67) | 0.219 | 0.48(0.35-0.60) | 0.722 | 0.64(0.48-0.80) | 0.095 | 0.53(0.43-0.63) | | 0.569 | 0.53(0.41-0.66) | 0.593 | 0.52(0.37-0.67) | 0.776 |
| S100A11 | 0.55(0.47-0.62) | 0.204 | 0.57(0.48-0.65) | 0.109 | 0.51(0.41-0.62) | 0.784 | 0.6(0.51-0.69) | 0.037 | 0.64(0.53-0.74) | 0.015 | 0.54(0.38-0.69) | 0.637 | 0.49(0.40-0.58) | | 0.881 | 0.49(0.38-0.60) | 0.838 | 0.5(0.36-0.64) | 0.984 |
| TNFSF13 | 0.52(0.45-0.60) | 0.580 | 0.57(0.48-0.66) | 0.122 | 0.55(0.44-0.66) | 0.376 | 0.6(0.50-0.70) | 0.040 | 0.61(0.50-0.73) | 0.056 | 0.58(0.42-0.75) | 0.318 | 0.54(0.44-0.64) | | 0.411 | 0.53(0.41-0.66) | 0.592 | 0.63(0.50-0.77) | 0.054 |
| ITGAV[1,2,5] | 0.57(0.50-0.65) | 0.051 | 0.56(0.47-0.65) | 0.190 | 0.59(0.49-0.69) | 0.080 | 0.57(0.47-0.66) | 0.191 | 0.53(0.40-0.66) | 0.640 | 0.63(0.50-0.76) | 0.054 | 0.58(0.49-0.67) | | 0.098 | 0.59(0.47-0.70) | 0.150 | 0.57(0.43-0.71) | 0.330 |
| DLL1 | 0.53(0.45-0.60) | 0.478 | 0.55(0.46-0.64) | 0.240 | 0.51(0.41-0.62) | 0.811 | 0.59(0.49-0.69) | 0.094 | 0.63(0.50-0.75) | 0.045 | 0.51(0.36-0.66) | 0.871 | 0.52(0.43-0.61) | | 0.687 | 0.51(0.40-0.62) | 0.849 | 0.53(0.40-0.66) | 0.679 |
| SMAD5 | 0.51(0.44-0.58) | 0.767 | 0.51(0.42-0.60) | 0.836 | 0.54(0.43-0.65) | 0.464 | 0.57(0.48-0.67) | 0.128 | 0.56(0.46-0.66) | 0.262 | 0.4(0.23-0.57) | 0.264 | 0.57(0.48-0.67) | | 0.111 | 0.53(0.41-0.65) | 0.584 | 0.63(0.50-0.75) | 0.049 |
| CA9 | 0.51(0.43-0.58) | 0.877 | 0.5(0.42-0.59) | 0.927 | 0.51(0.40-0.62) | 0.880 | 0.55(0.45-0.66) | 0.307 | 0.52(0.40-0.63) | 0.785 | 0.62(0.43-0.81) | 0.206 | 0.53(0.44-0.62) | | 0.493 | 0.51(0.39-0.62) | 0.911 | 0.56(0.44-0.68) | 0.308 |
| IL6[1,2,5] | 0.56(0.49-0.64) | 0.083 | 0.58(0.49-0.67) | 0.068 | 0.54(0.43-0.65) | 0.459 | 0.57(0.46-0.68) | 0.210 | 0.62(0.50-0.75) | 0.058 | 0.47(0.29-0.66) | 0.785 | 0.56(0.47-0.65) | | 0.169 | 0.55(0.44-0.66) | 0.400 | 0.58(0.45-0.71) | 0.224 |
| IFNGR1[2] | 0.52(0.45-0.59) | 0.570 | 0.55(0.46-0.64) | 0.302 | 0.52(0.41-0.62) | 0.760 | 0.59(0.50-0.69) | 0.056 | 0.62(0.50-0.74) | 0.050 | 0.55(0.41-0.68) | 0.502 | 0.53(0.44-0.62) | | 0.504 | 0.51(0.40-0.63) | 0.815 | 0.55(0.42-0.69) | 0.443 |
| GPC1[2] | 0.54(0.46-0.61) | 0.308 | 0.54(0.45-0.63) | 0.379 | 0.54(0.43-0.64) | 0.518 | 0.55(0.45-0.66) | 0.329 | 0.53(0.39-0.66) | 0.699 | 0.6(0.44-0.76) | 0.210 | 0.61(0.52-0.70) | | 0.017 | 0.6(0.49-0.71) | 0.076 | 0.62(0.49-0.75) | 0.076 |
| EPHA2 | 0.51(0.43-0.58) | 0.846 | 0.51(0.42-0.60) | 0.773 | 0.5(0.39-0.61) | 0.981 | 0.59(0.49-0.69) | 0.082 | 0.62(0.50-0.74) | 0.053 | 0.54(0.37-0.72) | 0.639 | 0.55(0.46-0.65) | | 0.237 | 0.58(0.46-0.69) | 0.196 | 0.53(0.40-0.66) | 0.678 |
| HGF[5] | 0.53(0.46-0.61) | 0.364 | 0.54(0.45-0.63) | 0.390 | 0.53(0.41-0.65) | 0.625 | 0.59(0.49-0.70) | 0.082 | 0.62(0.49-0.74) | 0.063 | 0.55(0.37-0.74) | 0.582 | 0.51(0.42-0.60) | | 0.849 | 0.53(0.42-0.63) | 0.601 | 0.48(0.33-0.63) | 0.839 |
| FGFBP1^2^ | 0.55(0.48-0.62) | 0.169 | 0.56(0.48-0.64) | 0.155 | 0.54(0.43-0.64) | 0.496 | 0.57(0.47-0.67) | 0.163 | 0.55(0.44-0.65) | 0.409 | 0.61(0.43-0.80) | 0.229 | 0.53(0.45-0.62) | | 0.420 | 0.57(0.47-0.68) | 0.180 | 0.51(0.40-0.63) | 0.842 |
| SEZ6L | 0.56(0.49-0.63) | 0.110 | 0.56(0.47-0.65) | 0.215 | 0.57(0.46-0.68) | 0.236 | 0.48(0.38-0.59) | 0.746 | 0.53(0.41-0.66) | 0.596 | 0.51(0.34-0.69) | 0.883 | 0.59(0.50-0.68) | | 0.045 | 0.58(0.46-0.69) | 0.193 | 0.61(0.48-0.75) | 0.095 |
| ADAMTS15[2] | 0.56(0.48-0.63) | 0.138 | 0.59(0.50-0.68) | 0.049 | 0.49(0.38-0.60) | 0.885 | 0.61(0.50-0.71) | 0.041 | 0.61(0.49-0.74) | 0.080 | 0.59(0.44-0.74) | 0.250 | 0.48(0.38-0.58) | | 0.673 | 0.57(0.45-0.69) | 0.243 | 0.54(0.39-0.69) | 0.593 |
| CD27 | 0.54(0.46-0.61) | 0.309 | 0.54(0.46-0.63) | 0.323 | 0.53(0.42-0.64) | 0.599 | 0.59(0.49-0.69) | 0.074 | 0.61(0.50-0.73) | 0.056 | 0.55(0.38-0.72) | 0.563 | 0.5(0.41-0.59) | | 0.987 | 0.51(0.40-0.63) | 0.809 | 0.48(0.35-0.62) | 0.816 |
| CD70[2] | 0.59(0.52-0.66) | 0.017 | 0.59(0.51-0.68) | 0.023 | 0.57(0.47-0.68) | 0.163 | 0.59(0.50-0.68) | 0.056 | 0.61(0.50-0.72) | 0.044 | 0.55(0.40-0.71) | 0.486 | 0.58(0.50-0.67) | | 0.063 | 0.58(0.48-0.69) | 0.133 | 0.58(0.45-0.72) | 0.206 |
| TXLNA | 0.51(0.43-0.58) | 0.838 | 0.56(0.47-0.64) | 0.207 | 0.56(0.46-0.67) | 0.240 | 0.49(0.39-0.60) | 0.902 | 0.55(0.43-0.67) | 0.399 | 0.61(0.44-0.77) | 0.198 | 0.52(0.43-0.61) | | 0.693 | 0.56(0.45-0.67) | 0.299 | 0.53(0.41-0.66) | 0.595 |
| KLK13[2] | 0.51(0.44-0.59) | 0.700 | 0.53(0.44-0.61) | 0.558 | 0.57(0.47-0.67) | 0.164 | 0.56(0.46-0.65) | 0.250 | 0.61(0.49-0.73) | 0.078 | 0.54(0.42-0.66) | 0.549 | 0.57(0.48-0.65) | | 0.134 | 0.55(0.45-0.64) | 0.367 | 0.59(0.46-0.73) | 0.186 |
| CEACAM5[2] | 0.58(0.51-0.65) | 0.028 | 0.57(0.49-0.66) | 0.099 | 0.59(0.49-0.69) | 0.078 | 0.56(0.47-0.65) | 0.218 | 0.54(0.43-0.65) | 0.503 | 0.4(0.26-0.55) | 0.204 | 0.6(0.51-0.69) | | 0.038 | 0.61(0.48-0.73) | 0.093 | 0.59(0.46-0.71) | 0.181 |
| RET[2] | 0.55(0.48-0.62) | 0.157 | 0.58(0.50-0.66) | 0.047 | 0.51(0.40-0.61) | 0.912 | 0.6(0.50-0.70) | 0.041 | 0.6(0.49-0.71) | 0.080 | 0.6(0.43-0.77) | 0.239 | 0.51(0.43-0.60) | | 0.750 | 0.57(0.47-0.67) | 0.193 | 0.55(0.43-0.67) | 0.391 |
| VEGFA[1,5] | 0.52(0.44-0.60) | 0.601 | 0.48(0.39-0.57) | 0.658 | 0.52(0.41-0.63) | 0.739 | 0.55(0.43-0.67) | 0.394 | 0.57(0.42-0.72) | 0.355 | 0.52(0.33-0.71) | 0.861 | 0.57(0.49-0.66) | | 0.094 | 0.6(0.50-0.70) | 0.048 | 0.54(0.40-0.68) | 0.555 |
| CTSV[1] | 0.5(0.43-0.58) | 0.916 | 0.55(0.46-0.64) | 0.293 | 0.56(0.45-0.67) | 0.270 | 0.52(0.42-0.63) | 0.647 | 0.51(0.39-0.63) | 0.860 | 0.55(0.36-0.74) | 0.610 | 0.53(0.43-0.62) | | 0.596 | 0.6(0.48-0.72) | 0.105 | 0.57(0.44-0.70) | 0.296 |
| CD207[2] | 0.5(0.43-0.57) | 0.970 | 0.53(0.45-0.62) | 0.474 | 0.54(0.43-0.65) | 0.450 | 0.53(0.43-0.63) | 0.537 | 0.51(0.40-0.62) | 0.916 | 0.6(0.43-0.77) | 0.268 | 0.53(0.44-0.62) | | 0.575 | 0.55(0.44-0.66) | 0.354 | 0.49(0.36-0.63) | 0.907 |
| NT5E | 0.51(0.43-0.58) | 0.881 | 0.53(0.44-0.62) | 0.506 | 0.53(0.43-0.63) | 0.579 | 0.54(0.44-0.64) | 0.481 | 0.5(0.37-0.62) | 0.964 | 0.6(0.44-0.76) | 0.241 | 0.54(0.45-0.63) | | 0.411 | 0.56(0.45-0.67) | 0.307 | 0.49(0.36-0.62) | 0.863 |
| CPE | 0.55(0.47-0.62) | 0.225 | 0.56(0.47-0.65) | 0.196 | 0.53(0.42-0.64) | 0.624 | 0.49(0.39-0.60) | 0.904 | 0.56(0.44-0.68) | 0.356 | 0.58(0.41-0.75) | 0.340 | 0.58(0.48-0.67) | | 0.107 | 0.56(0.44-0.68) | 0.316 | 0.59(0.46-0.73) | 0.164 |
| LY9[2] | 0.51(0.44-0.59) | 0.681 | 0.53(0.45-0.61) | 0.469 | 0.49(0.39-0.59) | 0.876 | 0.54(0.45-0.63) | 0.339 | 0.54(0.43-0.65) | 0.448 | 0.55(0.42-0.67) | 0.483 | 0.56(0.47-0.65) | | 0.192 | 0.59(0.49-0.70) | 0.073 | 0.51(0.38-0.65) | 0.833 |
| MIA | 0.5(0.43-0.58) | 0.915 | 0.52(0.44-0.61) | 0.589 | 0.53(0.42-0.63) | 0.636 | 0.54(0.44-0.64) | 0.410 | 0.49(0.36-0.61) | 0.819 | 0.59(0.43-0.75) | 0.258 | 0.54(0.45-0.63) | | 0.389 | 0.56(0.45-0.67) | 0.308 | 0.48(0.36-0.61) | 0.810 |
| TGFBR2[2] | 0.5(0.43-0.57) | 0.975 | 0.53(0.45-0.61) | 0.487 | 0.55(0.45-0.65) | 0.368 | 0.5(0.41-0.60) | 0.936 | 0.56(0.44-0.67) | 0.324 | 0.59(0.45-0.74) | 0.206 | 0.5(0.42-0.59) | | 0.912 | 0.51(0.40-0.61) | 0.915 | 0.52(0.39-0.65) | 0.780 |
| VEGFR3 | 0.5(0.43-0.58) | 0.906 | 0.52(0.44-0.61) | 0.575 | 0.53(0.42-0.63) | 0.632 | 0.54(0.44-0.64) | 0.428 | 0.49(0.36-0.61) | 0.844 | 0.59(0.43-0.75) | 0.265 | 0.54(0.45-0.63) | | 0.393 | 0.56(0.45-0.67) | 0.307 | 0.48(0.36-0.61) | 0.818 |
| KLK8 | 0.51(0.43-0.58) | 0.884 | 0.53(0.44-0.62) | 0.483 | 0.53(0.42-0.64) | 0.573 | 0.55(0.44-0.65) | 0.358 | 0.59(0.47-0.71) | 0.143 | 0.47(0.30-0.64) | 0.751 | 0.53(0.43-0.62) | | 0.568 | 0.52(0.41-0.63) | 0.718 | 0.53(0.39-0.68) | 0.634 |
| CYR61 | 0.52(0.45-0.60) | 0.561 | 0.51(0.43-0.60) | 0.758 | 0.53(0.42-0.65) | 0.557 | 0.53(0.43-0.63) | 0.534 | 0.51(0.41-0.62) | 0.790 | 0.56(0.38-0.73) | 0.504 | 0.56(0.47-0.65) | | 0.190 | 0.54(0.43-0.65) | 0.509 | 0.59(0.45-0.73) | 0.206 |
| TNFRSF19[2] | 0.51(0.43-0.58) | 0.883 | 0.51(0.42-0.59) | 0.892 | 0.52(0.41-0.63) | 0.682 | 0.58(0.48-0.67) | 0.137 | 0.59(0.47-0.71) | 0.149 | 0.55(0.40-0.70) | 0.535 | 0.57(0.48-0.66) | | 0.151 | 0.57(0.46-0.68) | 0.230 | 0.57(0.43-0.71) | 0.358 |
| ESM1 | 0.49(0.42-0.57) | 0.825 | 0.52(0.44-0.61) | 0.585 | 0.56(0.45-0.66) | 0.287 | 0.54(0.45-0.64) | 0.375 | 0.57(0.46-0.69) | 0.225 | 0.49(0.34-0.65) | 0.950 | 0.55(0.46-0.64) | | 0.313 | 0.48(0.36-0.60) | 0.798 | 0.59(0.46-0.72) | 0.187 |
| CD48[2] | 0.52(0.44-0.59) | 0.638 | 0.52(0.43-0.61) | 0.620 | 0.51(0.40-0.62) | 0.845 | 0.56(0.46-0.65) | 0.226 | 0.59(0.48-0.70) | 0.123 | 0.51(0.36-0.65) | 0.941 | 0.51(0.42-0.61) | | 0.804 | 0.53(0.41-0.65) | 0.593 | 0.49(0.34-0.63) | 0.851 |
| TLR3[2] | 0.52(0.45-0.59) | 0.577 | 0.49(0.41-0.57) | 0.802 | 0.53(0.43-0.64) | 0.523 | 0.52(0.43-0.61) | 0.641 | 0.52(0.42-0.61) | 0.761 | 0.59(0.43-0.74) | 0.266 | 0.52(0.43-0.61) | | 0.665 | 0.53(0.43-0.63) | 0.531 | 0.5(0.37-0.64) | 0.962 |
| LYPD3[2] | 0.53(0.46-0.60) | 0.441 | 0.49(0.41-0.58) | 0.846 | 0.56(0.45-0.66) | 0.279 | 0.5(0.40-0.60) | 0.979 | 0.55(0.44-0.66) | 0.399 | 0.58(0.44-0.73) | 0.270 | 0.55(0.46-0.64) | | 0.262 | 0.56(0.45-0.66) | 0.285 | 0.54(0.41-0.68) | 0.547 |
| TRAIL | 0.54(0.46-0.61) | 0.333 | 0.54(0.44-0.63) | 0.439 | 0.54(0.43-0.64) | 0.482 | 0.5(0.40-0.60) | 0.952 | 0.52(0.39-0.65) | 0.809 | 0.54(0.39-0.68) | 0.611 | 0.56(0.47-0.65) | | 0.188 | 0.58(0.46-0.70) | 0.171 | 0.54(0.40-0.67) | 0.593 |
| EGF[2] | 0.54(0.47-0.61) | 0.281 | 0.54(0.46-0.62) | 0.333 | 0.54(0.43-0.65) | 0.497 | 0.57(0.47-0.67) | 0.170 | 0.56(0.45-0.67) | 0.278 | 0.58(0.41-0.75) | 0.363 | 0.52(0.43-0.61) | | 0.686 | 0.52(0.42-0.63) | 0.669 | 0.51(0.38-0.65) | 0.859 |
| CDKN1A | 0.54(0.47-0.62) | 0.266 | 0.56(0.47-0.65) | 0.179 | 0.52(0.40-0.63) | 0.773 | 0.54(0.43-0.65) | 0.457 | 0.54(0.41-0.66) | 0.573 | 0.55(0.36-0.74) | 0.615 | 0.54(0.45-0.63) | | 0.346 | 0.58(0.47-0.69) | 0.149 | 0.5(0.36-0.63) | 0.961 |
| SYND1 | 0.52(0.45-0.59) | 0.573 | 0.52(0.44-0.61) | 0.589 | 0.52(0.41-0.63) | 0.758 | 0.57(0.47-0.66) | 0.182 | 0.56(0.44-0.67) | 0.315 | 0.58(0.42-0.74) | 0.339 | 0.51(0.42-0.60) | | 0.784 | 0.51(0.40-0.62) | 0.907 | 0.52(0.38-0.66) | 0.774 |
| VIM | 0.52(0.45-0.60) | 0.491 | 0.51(0.42-0.59) | 0.905 | 0.57(0.47-0.67) | 0.169 | 0.5(0.50-0.50) | 0.999 | 0.55(0.44-0.66) | 0.350 | 0.55(0.41-0.70) | 0.485 | 0.55(0.47-0.64) | | 0.219 | 0.53(0.43-0.64) | 0.530 | 0.58(0.46-0.70) | 0.207 |
| ITGB5[2] | 0.54(0.47-0.61) | 0.289 | 0.54(0.45-0.62) | 0.376 | 0.54(0.44-0.64) | 0.445 | 0.49(0.40-0.59) | 0.913 | 0.51(0.39-0.62) | 0.912 | 0.53(0.39-0.66) | 0.696 | 0.56(0.48-0.65) | | 0.156 | 0.58(0.47-0.69) | 0.160 | 0.55(0.42-0.68) | 0.489 |
| ANXA1 | 0.52(0.45-0.59) | 0.569 | 0.51(0.42-0.60) | 0.813 | 0.54(0.44-0.64) | 0.479 | 0.54(0.45-0.64) | 0.370 | 0.57(0.46-0.68) | 0.232 | 0.5(0.34-0.66) | 0.992 | 0.57(0.48-0.66) | | 0.118 | 0.58(0.47-0.69) | 0.170 | 0.56(0.44-0.68) | 0.345 |
| FCRLB | 0.5(0.43-0.58) | 0.906 | 0.53(0.44-0.62) | 0.505 | 0.53(0.43-0.64) | 0.543 | 0.53(0.43-0.63) | 0.551 | 0.5(0.38-0.61) | 0.942 | 0.58(0.41-0.74) | 0.366 | 0.53(0.44-0.62) | | 0.495 | 0.56(0.45-0.67) | 0.298 | 0.5(0.38-0.63) | 0.943 |
| SPARC | 0.51(0.44-0.58) | 0.803 | 0.49(0.40-0.57) | 0.785 | 0.51(0.39-0.62) | 0.925 | 0.55(0.45-0.66) | 0.324 | 0.56(0.44-0.69) | 0.330 | 0.54(0.36-0.71) | 0.696 | 0.56(0.47-0.64) | | 0.212 | 0.58(0.47-0.68) | 0.141 | 0.53(0.39-0.67) | 0.670 |
| PPY | 0.53(0.46-0.60) | 0.429 | 0.56(0.48-0.65) | 0.149 | 0.52(0.41-0.63) | 0.709 | 0.56(0.45-0.67) | 0.287 | 0.57(0.45-0.70) | 0.250 | 0.53(0.34-0.72) | 0.756 | 0.51(0.42-0.60) | | 0.852 | 0.56(0.44-0.67) | 0.320 | 0.55(0.42-0.68) | 0.431 |
| FURIN[2] | 0.5(0.43-0.58) | 0.898 | 0.52(0.43-0.61) | 0.610 | 0.52(0.41-0.64) | 0.711 | 0.51(0.41-0.61) | 0.851 | 0.56(0.44-0.67) | 0.341 | 0.57(0.40-0.75) | 0.415 | 0.5(0.40-0.59) | | 0.981 | 0.51(0.39-0.62) | 0.917 | 0.51(0.37-0.65) | 0.884 |
| ABL1[2] | 0.5(0.43-0.57) | 0.950 | 0.5(0.42-0.58) | 0.989 | 0.5(0.39-0.60) | 0.930 | 0.56(0.46-0.66) | 0.249 | 0.57(0.46-0.69) | 0.223 | 0.53(0.36-0.70) | 0.704 | 0.54(0.46-0.62) | | 0.352 | 0.56(0.47-0.66) | 0.201 | 0.51(0.38-0.64) | 0.853 |
| TCL1A | 0.51(0.43-0.58) | 0.891 | 0.53(0.44-0.62) | 0.481 | 0.53(0.42-0.64) | 0.549 | 0.54(0.44-0.64) | 0.488 | 0.51(0.39-0.63) | 0.815 | 0.57(0.41-0.74) | 0.386 | 0.54(0.44-0.63) | | 0.444 | 0.57(0.46-0.68) | 0.214 | 0.51(0.37-0.65) | 0.902 |
| FADD[2,5] | 0.56(0.48-0.63) | 0.134 | 0.55(0.47-0.64) | 0.202 | 0.56(0.45-0.67) | 0.316 | 0.57(0.47-0.66) | 0.171 | 0.57(0.46-0.68) | 0.189 | 0.56(0.39-0.73) | 0.514 | 0.55(0.46-0.64) | | 0.317 | 0.54(0.43-0.65) | 0.491 | 0.56(0.42-0.70) | 0.434 |
| LGALS1 | 0.51(0.44-0.58) | 0.816 | 0.5(0.41-0.59) | 0.980 | 0.52(0.41-0.63) | 0.715 | 0.53(0.43-0.63) | 0.554 | 0.57(0.45-0.70) | 0.263 | 0.54(0.39-0.70) | 0.579 | 0.54(0.45-0.63) | | 0.394 | 0.56(0.46-0.67) | 0.222 | 0.51(0.37-0.64) | 0.940 |
| CRNN[2] | 0.52(0.45-0.59) | 0.605 | 0.54(0.45-0.63) | 0.433 | 0.51(0.41-0.60) | 0.911 | 0.49(0.39-0.60) | 0.912 | 0.53(0.40-0.66) | 0.642 | 0.57(0.43-0.72) | 0.340 | 0.54(0.45-0.63) | | 0.404 | 0.54(0.42-0.66) | 0.494 | 0.53(0.41-0.66) | 0.585 |
| MICA, MICB | 0.52(0.45-0.60) | 0.514 | 0.56(0.47-0.65) | 0.188 | 0.53(0.42-0.63) | 0.604 | 0.51(0.41-0.62) | 0.788 | 0.55(0.41-0.68) | 0.509 | 0.54(0.38-0.71) | 0.618 | 0.53(0.45-0.62) | | 0.473 | 0.57(0.47-0.67) | 0.181 | 0.52(0.39-0.64) | 0.775 |
| FASLG[2] | 0.53(0.45-0.60) | 0.445 | 0.56(0.47-0.65) | 0.202 | 0.51(0.40-0.62) | 0.809 | 0.51(0.41-0.61) | 0.814 | 0.54(0.43-0.66) | 0.439 | 0.55(0.38-0.71) | 0.574 | 0.54(0.45-0.64) | | 0.393 | 0.57(0.44-0.69) | 0.276 | 0.49(0.35-0.63) | 0.922 |
| WIF1[2] | 0.5(0.42-0.57) | 0.916 | 0.52(0.43-0.60) | 0.652 | 0.52(0.41-0.62) | 0.722 | 0.55(0.46-0.64) | 0.270 | 0.55(0.44-0.65) | 0.416 | 0.56(0.42-0.71) | 0.396 | 0.53(0.44-0.62) | | 0.482 | 0.5(0.39-0.61) | 0.960 | 0.57(0.44-0.70) | 0.300 |
| TGFA[5] | 0.51(0.44-0.58) | 0.797 | 0.54(0.45-0.62) | 0.400 | 0.47(0.36-0.58) | 0.589 | 0.55(0.46-0.65) | 0.275 | 0.56(0.45-0.68) | 0.253 | 0.54(0.36-0.71) | 0.684 | 0.52(0.44-0.61) | | 0.587 | 0.51(0.40-0.62) | 0.839 | 0.57(0.44-0.70) | 0.285 |
| IGF1R | 0.5(0.42-0.57) | 0.957 | 0.54(0.45-0.63) | 0.356 | 0.57(0.46-0.67) | 0.219 | 0.51(0.40-0.61) | 0.923 | 0.54(0.42-0.67) | 0.489 | 0.56(0.40-0.73) | 0.446 | 0.51(0.41-0.60) | | 0.876 | 0.54(0.42-0.66) | 0.498 | 0.57(0.43-0.70) | 0.318 |
| ERBB2[2] | 0.53(0.45-0.60) | 0.496 | 0.54(0.45-0.62) | 0.435 | 0.51(0.40-0.62) | 0.833 | 0.48(0.38-0.58) | 0.703 | 0.5(0.38-0.62) | 0.980 | 0.56(0.39-0.73) | 0.501 | 0.53(0.44-0.62) | | 0.528 | 0.57(0.45-0.68) | 0.255 | 0.48(0.34-0.63) | 0.830 |
| S100A4[2] | 0.53(0.45-0.60) | 0.506 | 0.51(0.42-0.60) | 0.843 | 0.55(0.44-0.66) | 0.379 | 0.56(0.46-0.66) | 0.273 | 0.55(0.43-0.67) | 0.387 | 0.56(0.39-0.73) | 0.470 | 0.5(0.41-0.59) | | 0.970 | 0.53(0.41-0.65) | 0.637 | 0.54(0.40-0.68) | 0.566 |
| METAP2 | 0.54(0.46-0.61) | 0.343 | 0.56(0.47-0.65) | 0.190 | 0.5(0.39-0.62) | 0.959 | 0.55(0.45-0.65) | 0.293 | 0.56(0.45-0.68) | 0.285 | 0.54(0.37-0.70) | 0.682 | 0.52(0.43-0.62) | | 0.632 | 0.55(0.44-0.67) | 0.362 | 0.52(0.37-0.66) | 0.821 |
| LYN | 0.53(0.46-0.60) | 0.366 | 0.55(0.46-0.63) | 0.296 | 0.49(0.39-0.58) | 0.777 | 0.53(0.43-0.63) | 0.574 | 0.56(0.43-0.68) | 0.352 | 0.53(0.38-0.67) | 0.730 | 0.54(0.45-0.62) | | 0.405 | 0.53(0.43-0.64) | 0.526 | 0.54(0.42-0.66) | 0.528 |
| CEACAM1[2] | 0.52(0.44-0.59) | 0.636 | 0.5(0.41-0.58) | 0.948 | 0.54(0.43-0.65) | 0.484 | 0.52(0.42-0.62) | 0.744 | 0.52(0.40-0.64) | 0.753 | 0.51(0.35-0.68) | 0.885 | 0.52(0.42-0.61) | | 0.693 | 0.51(0.40-0.63) | 0.851 | 0.56(0.41-0.70) | 0.443 |
| CD160 | 0.53(0.46-0.60) | 0.439 | 0.52(0.43-0.60) | 0.713 | 0.55(0.45-0.65) | 0.361 | 0.54(0.45-0.63) | 0.399 | 0.55(0.44-0.65) | 0.389 | 0.53(0.36-0.70) | 0.722 | 0.52(0.43-0.61) | | 0.668 | 0.51(0.40-0.62) | 0.852 | 0.56(0.44-0.67) | 0.345 |
| GZMH | 0.53(0.46-0.60) | 0.440 | 0.52(0.44-0.60) | 0.676 | 0.54(0.45-0.63) | 0.371 | 0.5(0.41-0.59) | 0.981 | 0.51(0.40-0.62) | 0.832 | 0.52(0.37-0.67) | 0.756 | 0.55(0.47-0.63) | | 0.253 | 0.54(0.44-0.64) | 0.412 | 0.55(0.45-0.66) | 0.327 |
| ICOSLG[2,3] | 0.53(0.45-0.60) | 0.481 | 0.52(0.43-0.60) | 0.737 | 0.54(0.43-0.66) | 0.451 | 0.54(0.43-0.64) | 0.483 | 0.54(0.41-0.67) | 0.549 | 0.47(0.29-0.64) | 0.692 | 0.52(0.43-0.61) | | 0.690 | 0.49(0.38-0.61) | 0.929 | 0.55(0.40-0.70) | 0.513 |
| SCAMP3[1] | 0.51(0.44-0.59) | 0.697 | 0.53(0.45-0.62) | 0.460 | 0.51(0.40-0.62) | 0.849 | 0.51(0.42-0.61) | 0.781 | 0.55(0.44-0.66) | 0.397 | 0.55(0.38-0.71) | 0.558 | 0.51(0.42-0.61) | | 0.747 | 0.52(0.41-0.62) | 0.761 | 0.51(0.37-0.65) | 0.860 |
| GPNMB | 0.51(0.43-0.58) | 0.841 | 0.51(0.42-0.60) | 0.815 | 0.5(0.39-0.62) | 0.950 | 0.51(0.40-0.61) | 0.900 | 0.52(0.39-0.64) | 0.807 | 0.55(0.38-0.72) | 0.587 | 0.51(0.42-0.60) | | 0.858 | 0.53(0.43-0.64) | 0.528 | 0.52(0.38-0.67) | 0.749 |
| SCF[1,2,5] | 0.54(0.46-0.61) | 0.319 | 0.54(0.45-0.63) | 0.411 | 0.54(0.43-0.64) | 0.489 | 0.53(0.43-0.63) | 0.594 | 0.53(0.40-0.66) | 0.675 | 0.53(0.39-0.67) | 0.709 | 0.55(0.45-0.64) | | 0.340 | 0.55(0.43-0.66) | 0.428 | 0.54(0.40-0.69) | 0.546 |
| AREG | 0.52(0.45-0.60) | 0.526 | 0.53(0.44-0.61) | 0.573 | 0.52(0.41-0.63) | 0.696 | 0.52(0.42-0.62) | 0.711 | 0.54(0.41-0.66) | 0.577 | 0.49(0.32-0.66) | 0.917 | 0.53(0.44-0.62) | | 0.559 | 0.52(0.41-0.63) | 0.773 | 0.54(0.40-0.68) | 0.568 |
| CXL17 | 0.49(0.42-0.57) | 0.870 | 0.5(0.41-0.58) | 0.935 | 0.51(0.40-0.62) | 0.864 | 0.51(0.40-0.62) | 0.850 | 0.5(0.38-0.62) | 0.951 | 0.53(0.34-0.73) | 0.724 | 0.52(0.43-0.61) | | 0.689 | 0.5(0.39-0.61) | 0.951 | 0.54(0.40-0.67) | 0.595 |

p = p-value

**References**

1. Leandersson P, Åkesson A, Hedenfalk I, Malander S, Borgfeldt C. A multiplex biomarker assay improves the diagnostic performance of HE4 and CA125 in ovarian tumor patients. PloS One. 2020;15(10):e0240418.

2. Skubitz APN, Boylan KLM, Geschwind K, Cao Q, Starr TK, Geller MA, et al. Simultaneous Measurement of 92 Serum Protein Biomarkers for the Development of a Multiprotein Classifier for Ovarian Cancer Detection. Cancer Prev Res Phila Pa. 2019;12(3):171–84.

3. Enroth S, Berggrund M, Lycke M, Broberg J, Lundberg M, Assarsson E, et al. High throughput proteomics identifies a high-accuracy 11 plasma protein biomarker signature for ovarian cancer. Commun Biol. 2019;2(1):1–12.

4. Enroth S, Berggrund M, Lycke M, Lundberg M, Assarsson E, Olovsson M, et al. A two-step strategy for identification of plasma protein biomarkers for endometrial and ovarian cancer. Clin Proteomics. 2018;15(1):38.

5. Boylan KLM, Geschwind K, Koopmeiners JS, Geller MA, Starr TK, Skubitz APN. A multiplex platform for the identification of ovarian cancer biomarkers. Clin Proteomics. 2017;14:34.
